# Editorial: Advances in neuromodulation for chronic pain: mechanisms and clinical implications

**DOI:** 10.3389/fpain.2026.1926081

**Published:** 2026-07-21

**Authors:** Hong Jiang, Linlin Sun, Fengyu Liu, Hong Jiang

**Affiliations:** 1 Neuroscience Research Institute and Department of Neurobiology, School of Basic Medical Sciences, Key Laboratory for Neuroscience, Ministry of Education/National Health Commission, Peking University, Beijing, China; 2 Beijing Life Science Academy, Beijing, China; 3 Department of Neurology, Peking University People’s Hospital, Beijing, China

**Keywords:** acupuncture, chronic pain, clinical translation, neuromodulation, non-invasive brain stimulation

Chronic pain remains one of the most challenging conditions in modern medicine, not only because of its high prevalence and socioeconomic burden, but also because it represents a dynamic disorder of the nervous system rather than a simple extension of peripheral injury. Persistent nociceptive input, maladaptive neuroplasticity, neuroinflammation, impaired descending inhibition, and affective-cognitive modulation together contribute to the transition from acute to chronic pain. These mechanisms help explain why conventional pharmacological approaches often provide incomplete relief, are limited by adverse effects, and may fail to address the neural processes that sustain pain chronicity. Against this background, neuromodulation has emerged as a promising therapeutic strategy that aims to reshape pain-processing pathways rather than merely suppress symptoms.

The research topic *Advances in Neuromodulation for Chronic Pain: Mechanisms and Clinical Implications* brings together five articles that collectively illustrate the breadth of this rapidly developing field. The contributions span non-invasive and invasive or device-based approaches, including electroacupuncture, transcranial magnetic stimulation, transcranial direct current stimulation, low-intensity pulsed ultrasound, and high-frequency spinal cord stimulation. Although these modalities differ in their biophysical principles, stimulation targets, and clinical contexts, they share a common conceptual foundation: chronic pain can be modified by intervening at peripheral, spinal, cortical, and network levels of the nervous system.

A key theme emerging from this Research Topic is that neuromodulation should not be viewed as a uniform analgesic intervention, but rather as a set of mechanism-informed strategies that may act on distinct pain generators. In their narrative review, Zhu et al. discuss electroacupuncture for temporomandibular disorder-related pain, a condition characterized by joint dysfunction, masticatory muscle involvement, inflammation, and central sensitization. The authors synthesize clinical evidence suggesting that electroacupuncture may alleviate pain and improve functional and psychological outcomes, while also highlighting possible mechanisms involving activation of sensory afferents, enhanced local blood flow, tissue repair, endogenous analgesic systems, and immunomodulation. Importantly, the review also points to a recurring challenge in the field: encouraging clinical signals must be interpreted in light of heterogeneous protocols, variable patient populations, and the need for standardized stimulation parameters.

Two articles focus on brain stimulation approaches for chronic craniofacial pain and headache disorders. Lin et al. review non-invasive brain stimulation for neuropathic orofacial pain, emphasizing repetitive transcranial magnetic stimulation and transcranial direct current stimulation. Their synthesis highlights the primary motor cortex as a major target for analgesic stimulation and raises the important possibility that pain phenotype may influence treatment response. For example, paroxysmal and persistent pain states may differ in the degree of central sensitization, descending inhibitory dysfunction, and cortical excitability changes, which could shape responsiveness to rTMS or tDCS. This phenotype-based perspective is particularly valuable because it moves the field beyond the question of whether stimulation works, toward the more clinically actionable question of which patients are most likely to benefit.

Xiao et al. further extend this mechanistic framework in their review of electrophysiological indicators in transcranial magnetic stimulation treatment for medication-overuse headache. Medication-overuse headache is closely associated with central sensitization, habituation deficits, altered cortical silent period duration, and abnormal pre-activation levels. By discussing these measurable electrophysiological features, the authors underscore the potential value of objective biomarkers in guiding neuromodulation. Such biomarkers may help identify abnormal cortical excitability, monitor treatment response, and eventually support individualized stimulation protocols. This is an important direction for the field, because clinical pain scores alone may not fully capture the neural changes induced by neuromodulatory therapies.

The topic also includes clinical evidence for peripheral or regionally targeted physical neuromodulation. Huang et al. report a randomized controlled trial comparing low-intensity pulsed ultrasound with tamsulosin in patients with chronic prostatitis type IIIb/chronic pelvic pain syndrome. Both interventions improved symptoms, but low-intensity pulsed ultrasound showed greater benefit for pain relief, whereas tamsulosin had stronger effects on urinary symptoms. This distinction is clinically informative because chronic pelvic pain syndrome is a multidimensional disorder involving pelvic discomfort, urinary symptoms, sexual dysfunction, inflammation, and psychosocial burden. The findings suggest that physical neuromodulation may be particularly relevant for pain-dominant phenotypes and may complement pharmacological strategies that target other symptom domains.

Finally, Gupta et al. explore an important practical dimension of implantable neuromodulation: how stimulation dosing can be optimized without sacrificing clinical benefit. Their study of extreme pulse dosing in 10 kHz spinal cord stimulation for chronic back or leg pain suggests that some patients may maintain pain relief and quality-of-life benefits with markedly reduced stimulation duty cycles. Beyond improving device convenience and reducing recharge burden, pulse dosing raises broader mechanistic questions about the minimum neural input required to sustain analgesia, the role of intermittent vs. continuous stimulation, and whether reduced dosing might mitigate habituation to therapy over time.

Taken together, these five articles highlight several priorities for the next stage of chronic pain neuromodulation research. First, stimulation protocols require greater standardization, including dose, frequency, duration, anatomical targeting, and treatment schedules. Second, patient stratification should become central to trial design. Pain location, phenotype, duration, sensory profile, emotional comorbidity, and evidence of central sensitization may all influence therapeutic outcomes. Third, mechanistic studies should be integrated into clinical trials wherever possible. Electrophysiology, quantitative sensory testing, neuroimaging, inflammatory markers, and autonomic or behavioral readouts may provide complementary insight into how neuromodulation changes pain-processing networks. Fourth, long-term follow-up is essential, as chronic pain treatment must be judged not only by short-term analgesia but also by durability, functional recovery, safety, adherence, and quality of life.

In conclusion, this research topic reflects a broader shift in chronic pain therapy from symptom suppression toward nervous-system modulation. The included articles demonstrate that neuromodulatory approaches can act across multiple biological scales, from local tissue inflammation and peripheral afferent signaling to cortical excitability, descending pain control, and spinal network modulation. While important questions remain regarding mechanisms, patient selection, and optimal protocols, the field is moving toward more precise, personalized, and mechanism-based interventions. Continued collaboration among clinicians, neuroscientists, engineers, and rehabilitation specialists will be essential for translating these advances into durable and accessible therapies for patients living with chronic pain.

